# Supplementary material for: High fat diet ameliorates mitochondrial cardiomyopathy in CHCHD10 mutant mice
Source: EMBO Mol Med. 2024 May 9;16(6):8. doi: 10.1038/s44321-024-00067-5 (PMC11178915; doi:10.1038/s44321-024-00067-5)
Supplement: Supplementary file 1 — Appendix [file 44321_2024_67_MOESM1_ESM.pdf]

High fat diet ameliorates mitochondrial cardiomyopathy in CHCHD10 mutant mice

Appendix

Contents

Appendix Figures

Appendix Figure S1: Echocardiographic measurements in WT and Het male mice on CD or HFD 2

Appendix Figure S2: Metabolic and behavioral analyses 4

Appendix Figure S3: Blood chemistry 5

Appendix Figure S4: Heart lipidomic analyses 6

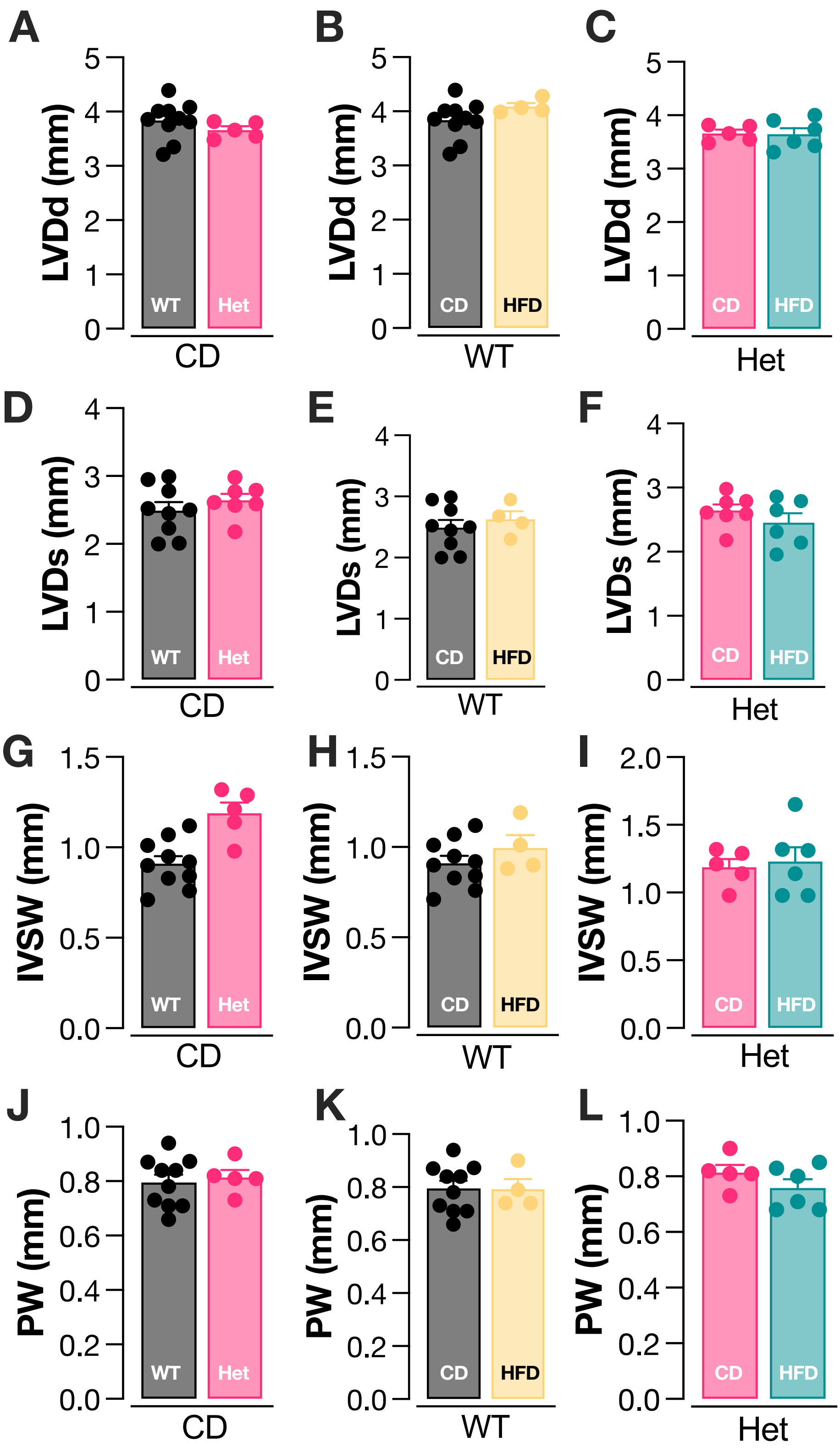
A
B
C
LVDd (mm)
WT
Het
CD
HFD
CD
WT
Het
D
E
F
LVDs (mm)
G
H
I
IVSW (mm)
J
K
L
PW (mm)

Appendix Figure S1. Echocardiographic measurements in WT and Het male mice on CD or HFD. (A-C) LV internal diameter end diastole (LVDd); (D-F) LV internal diameter end systole (LVDs); (G-I) interventricular septum wall (IVSW); (J-L) Posterior Wall (PW).

Data information: In panels A-L, WT CD (n = 10 mice), Het CD (n = 5 mice), WT HFD (n = 4 mice), Het HFD (n = 6 mice). Data are expressed as mean ± SEM.

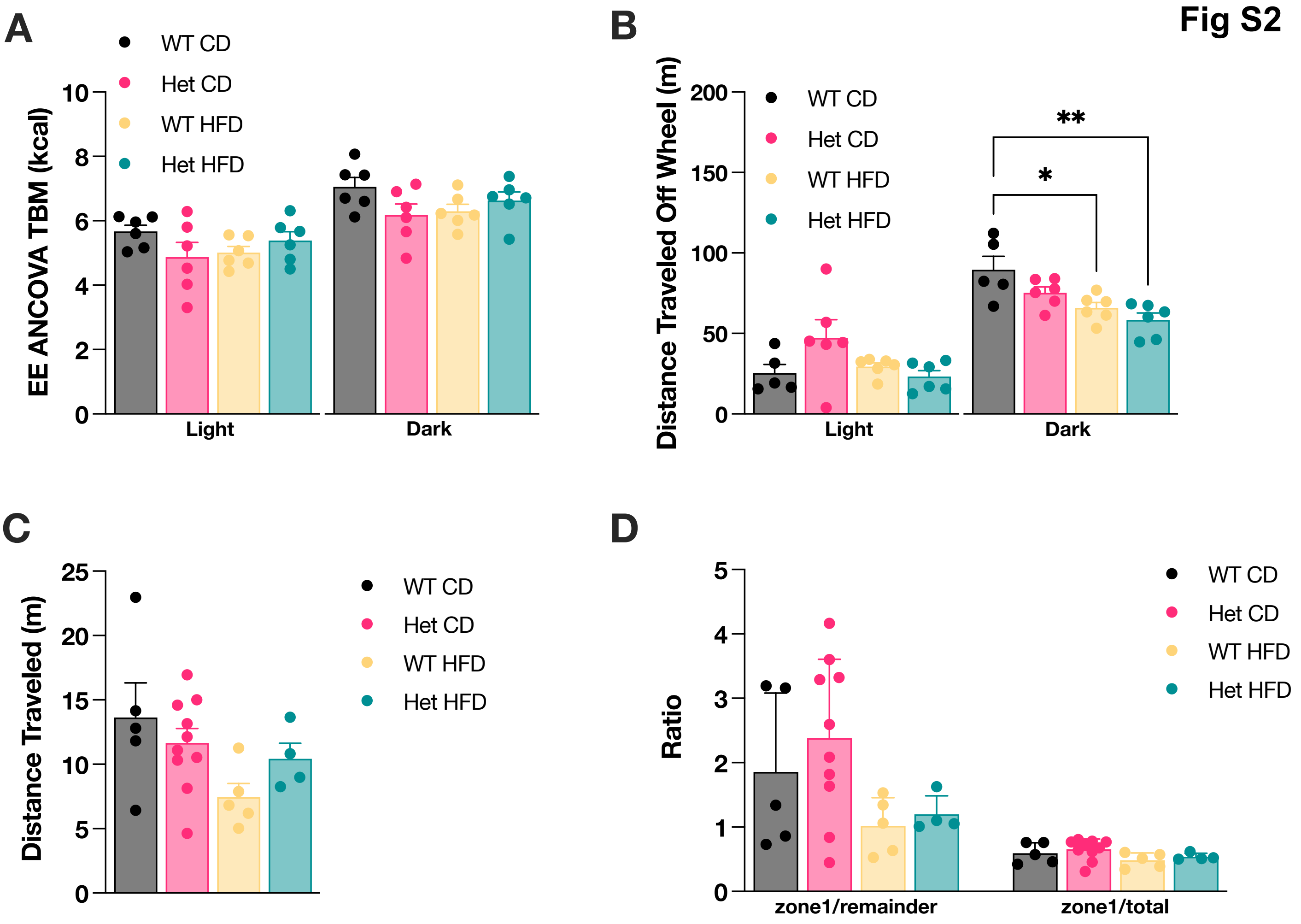

Appendix Figure S2. **Metabolic and behavioral analyses. A**) Energy expenditure (EE) during the light and dark cycle of mice in the Promethion metabolic phenotyping cage without a wheel. The calculated energy expenditure was adjusted to total body mass. **B**) Distance traveled in the cage, off the wheel, when wheels were present in the cages. **C**) Ambulatory distance in 30 minutes of recording in an open field test system. **D**) Ratio of ambulatory time spent in a specified zone of the recording cage (zone 1) to the remainder of the cage, and versus total ambulatory time.

In A-B, groups were comprised of males. WT CD (n = 6 mice), Het CD (n = 6 mice), WT HFD (n = 6 mice), Het HFD (n = 6 mice). For C-D, groups were comprised of females. WT CD (n = 5 mice), Het CD (n = 10 mice), WT HFD (n = 5 mice), Het HFD (n = 4 mice). Statistical significance was determined by one-way ANOVA with Tukey's correction. * $p < 0.05$, ** $p < 0.01$. Data are expressed as mean ± SEM.

Fig S3

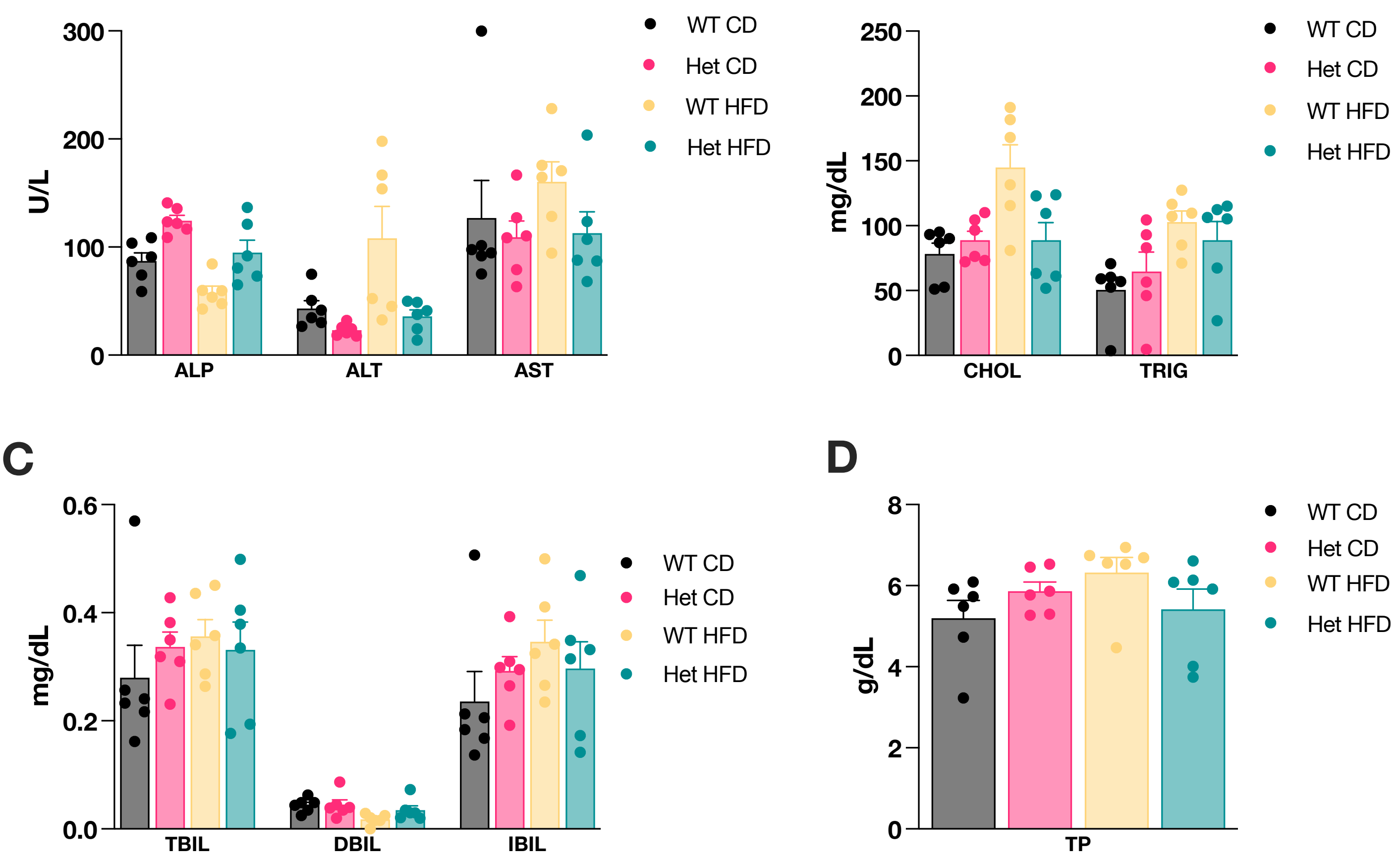

Appendix Figure S3. **Blood chemistry**. **A**) Serum Alkaline phosphatase (ALP), aspartate transaminase (AST), and alanine transaminase (ALT) levels in Het and WT mice on CD or HFD at P250. **B**) Serum cholesterol (CHOL) and triglyceride (TRIG) levels. **C**) Total bilirubin (TBIL), direct bilirubin (DBIL), indirect bilirubin (IBIL). **D**) Serum total protein content.

Data information: In A-D, WT CD (n = 6 mice), Het CD (n = 6 mice), WT HFD (n = 6 mice), Het HFD (n = 6 mice). Statistical significance was determined by one-way ANOVA with Tukey's correction. Data are expressed as mean ± SEM.

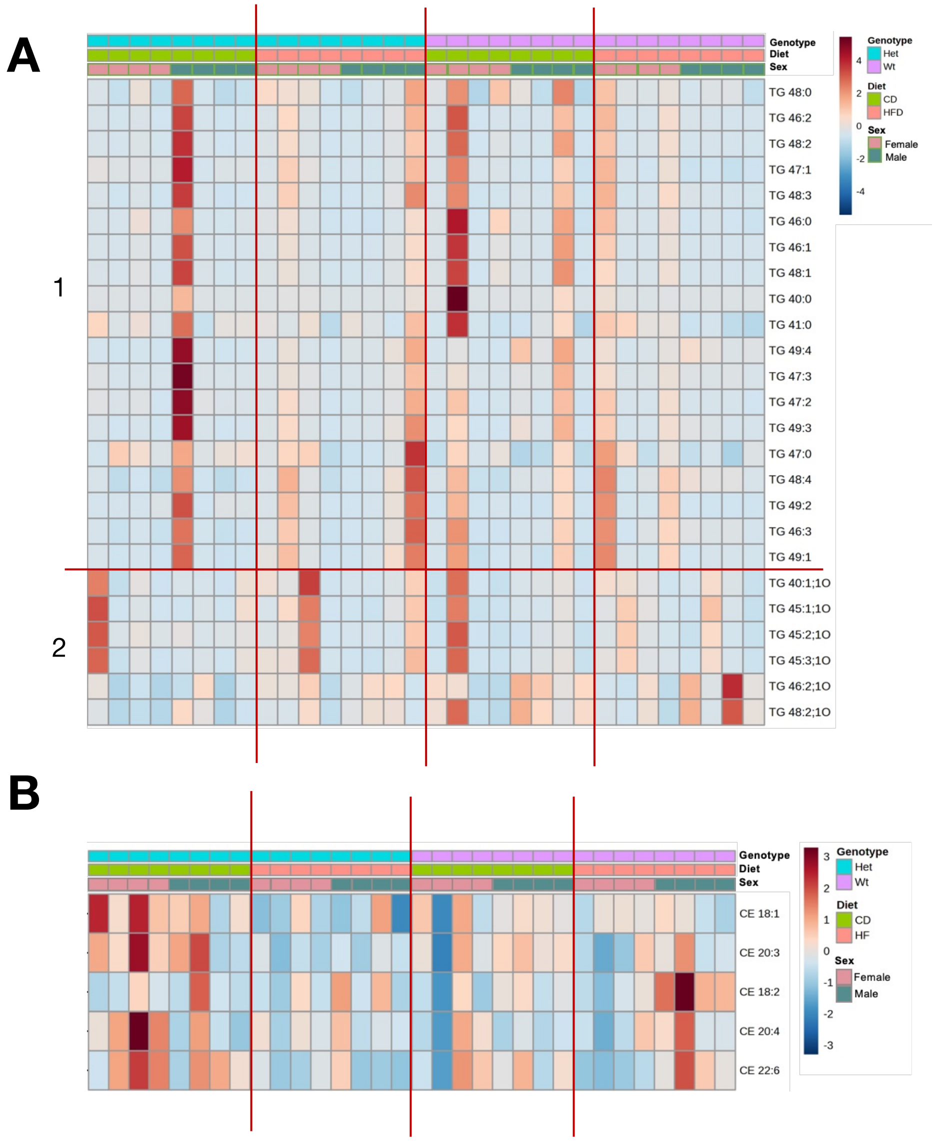

Appendix Figure S4. **Heart lipidomic analyses**. **A**) Heatmap of triglycerides in Het and WT heart on CD or HFD. **B**) Heatmap of cholesterol esters in Het and WT heart on CD or HFD.

Data information: In A and B, WT CD (n = 8 mice), Het CD (n = 8 mice), WT HFD (n = 8 mice), Het HFD (n = 8 mice). Equal numbers of males and females were used. The red lines and the numbers on the left denote different groups of metabolites that change based on genotype, diet, and sex.
