# Supplementary figures and images for: High fat diet ameliorates mitochondrial cardiomyopathy in CHCHD10 mutant mice
Source: EMBO Mol Med. 2024 May 9;16(6):8. doi: 10.1038/s44321-024-00067-5 (PMC11178915; doi:10.1038/s44321-024-00067-5)

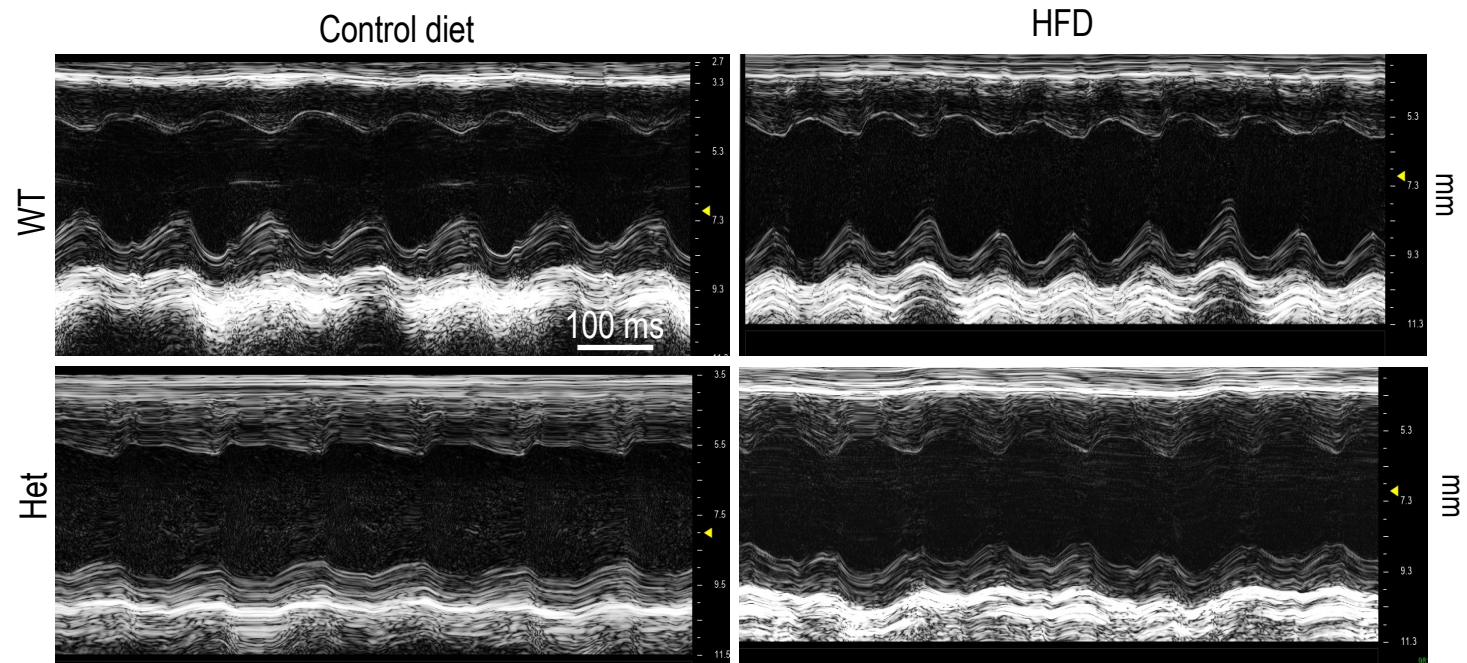

Supplement: Supplementary file 2 — Source data Fig. 1 [file 44321_2024_67_MOESM2_ESM.zip › Figure 1/1D/Fig 1D.pdf]

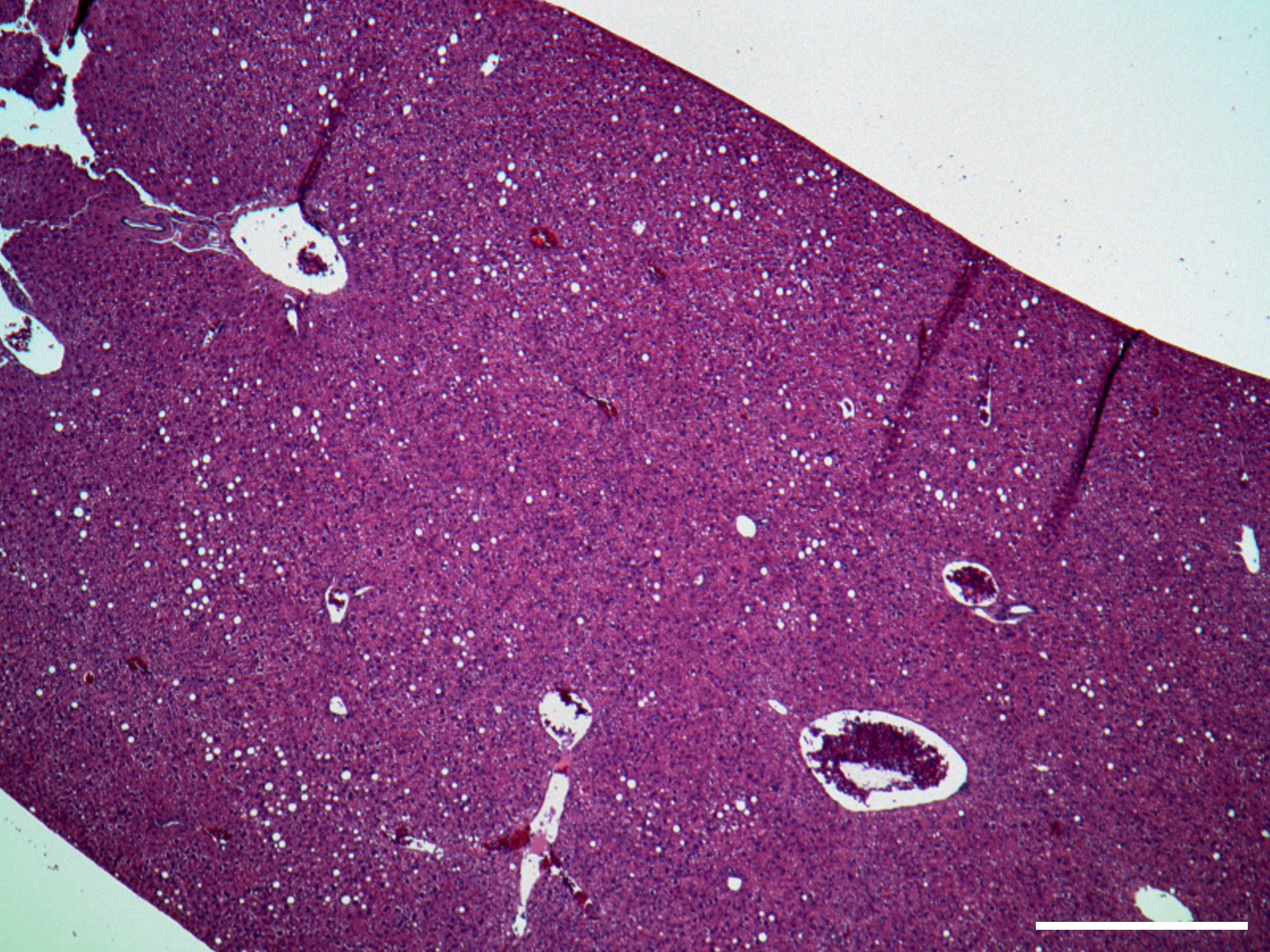

Supplement: Supplementary file 4 — Source data Fig. 3 [file 44321_2024_67_MOESM4_ESM.zip › Figure 3/3E/Het HFD 500 um.tif]

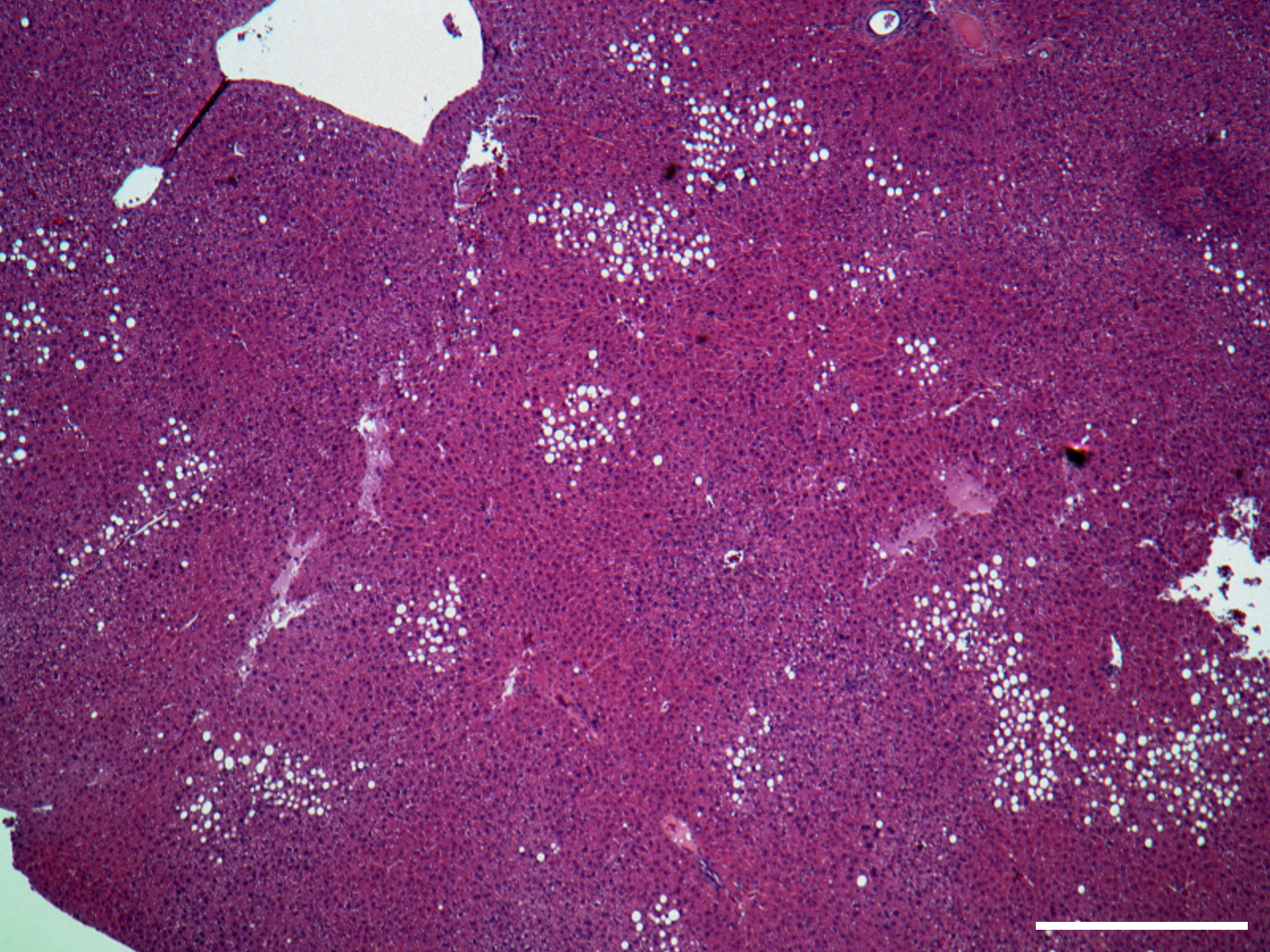

Supplement: Supplementary file 4 — Source data Fig. 3 [file 44321_2024_67_MOESM4_ESM.zip › Figure 3/3E/WT HFD 500 um.tif]

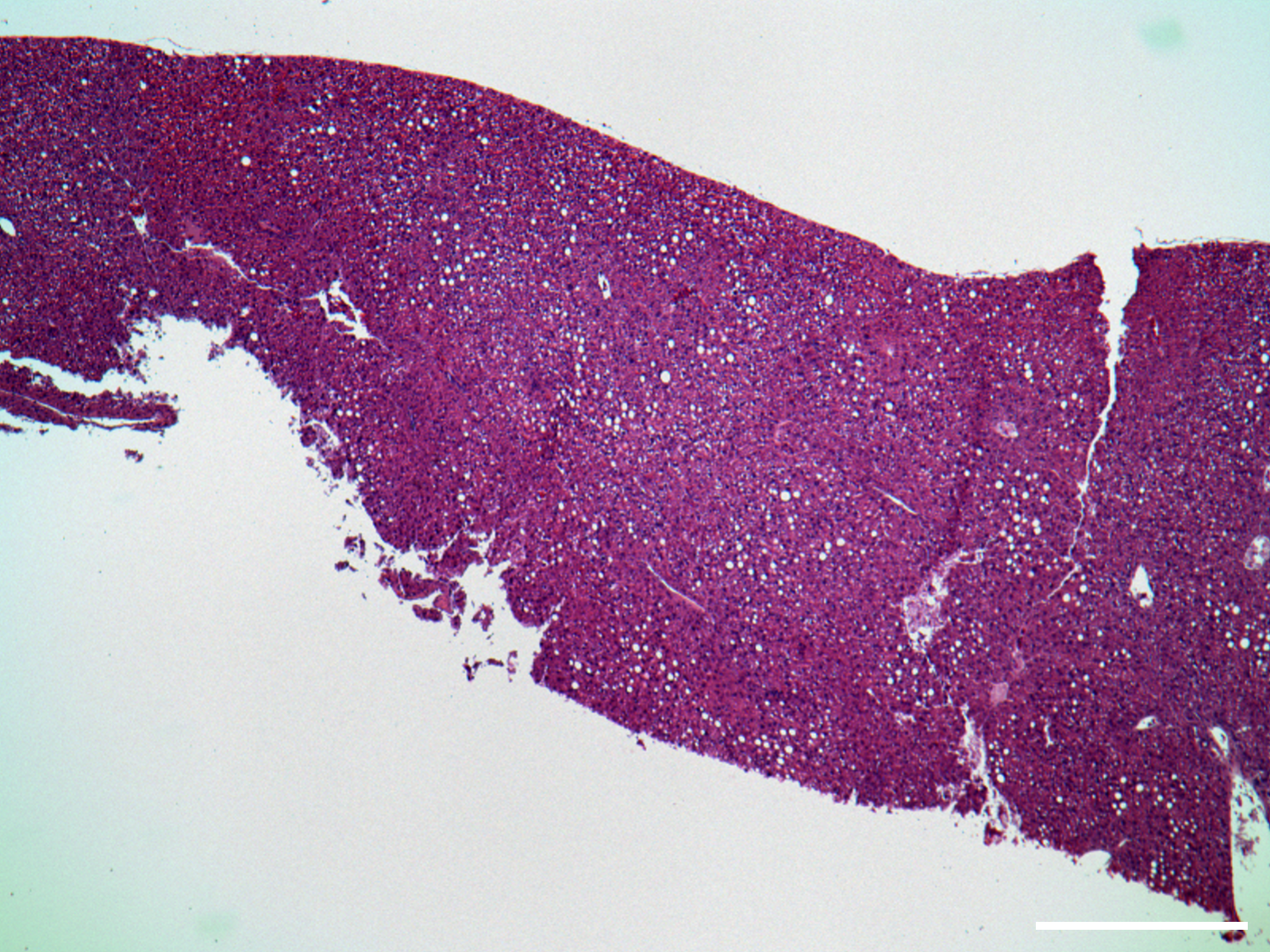

Supplement: Supplementary file 4 — Source data Fig. 3 [file 44321_2024_67_MOESM4_ESM.zip › Figure 3/3E/WT CD 500 um.tif]

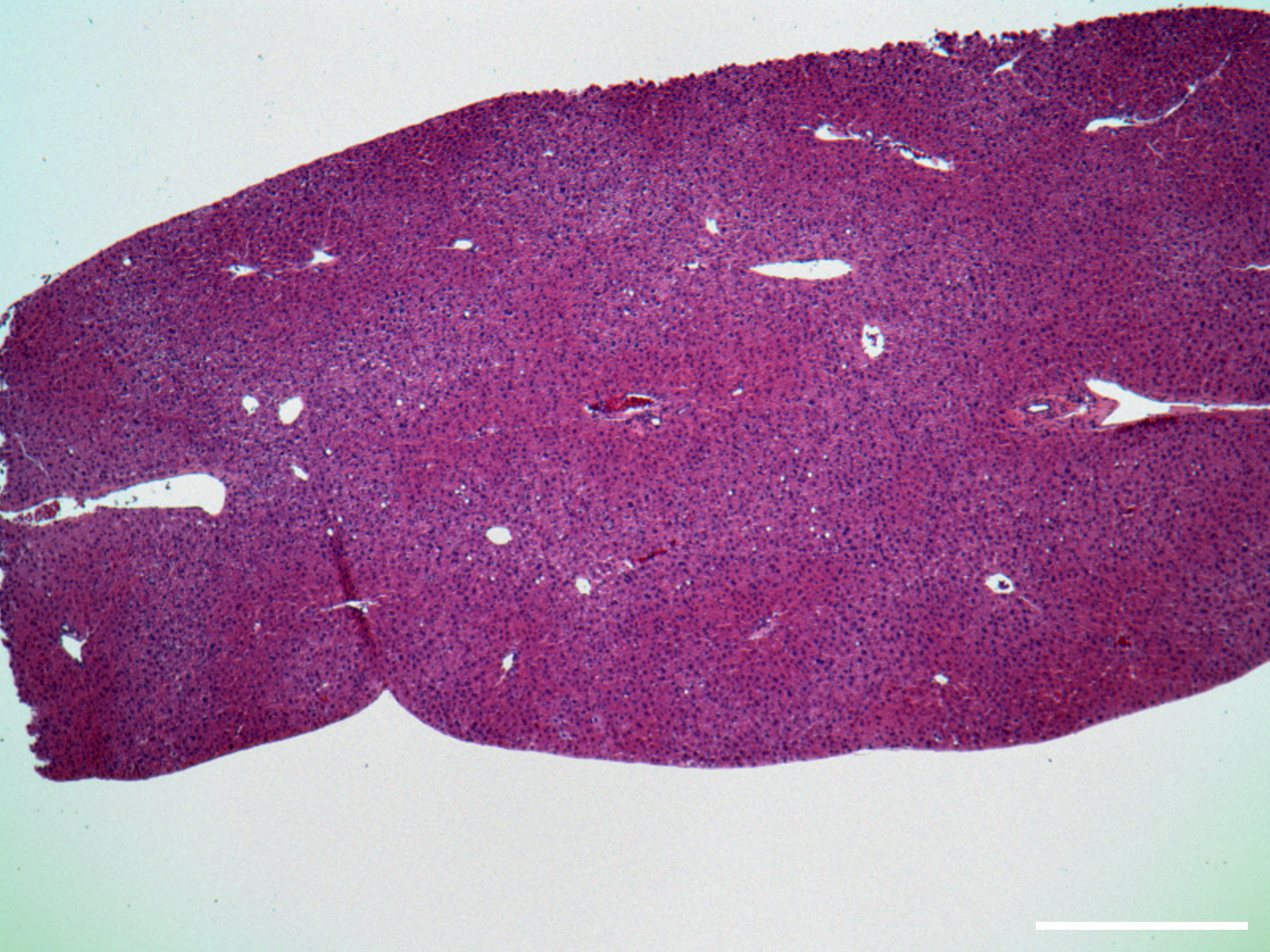

Supplement: Supplementary file 4 — Source data Fig. 3 [file 44321_2024_67_MOESM4_ESM.zip › Figure 3/3E/Het CD 500 um.tif]

Figure 8F

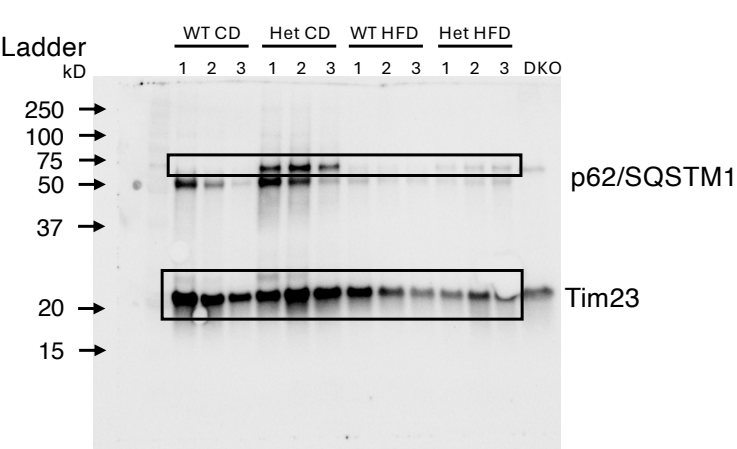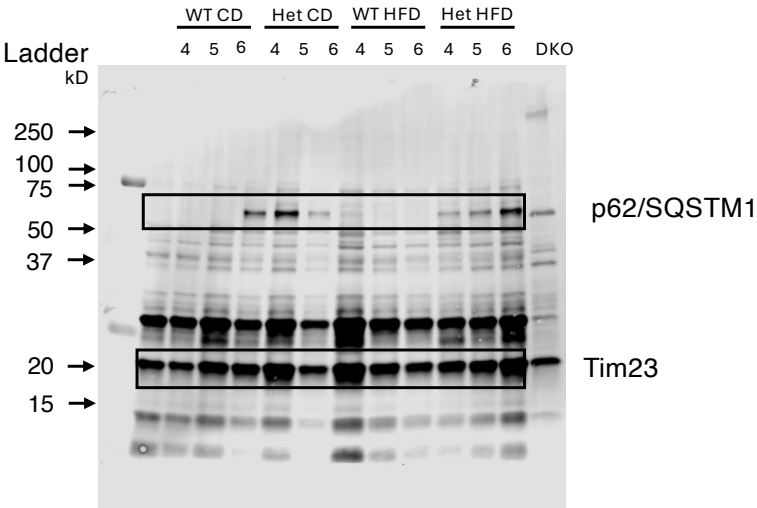

Supplement: Supplementary file 6 — Source data Fig. 8 [file 44321_2024_67_MOESM6_ESM.zip › Figure 8/8F-G/8F.pdf]

Figure 8N

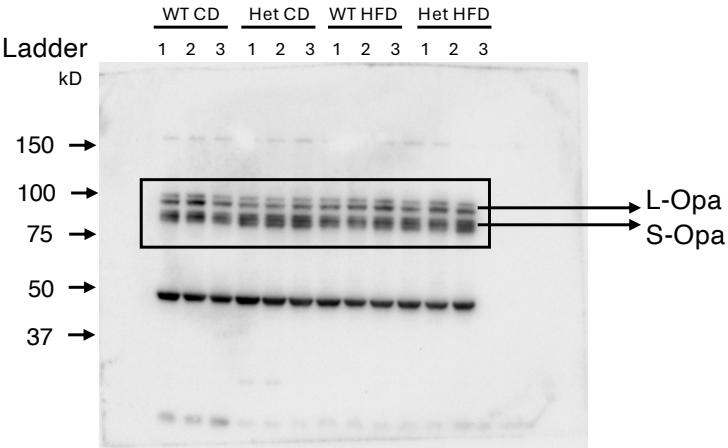

Supplement: Supplementary file 6 — Source data Fig. 8 [file 44321_2024_67_MOESM6_ESM.zip › Figure 8/8N-Q/8N.pdf]

Figure 8P

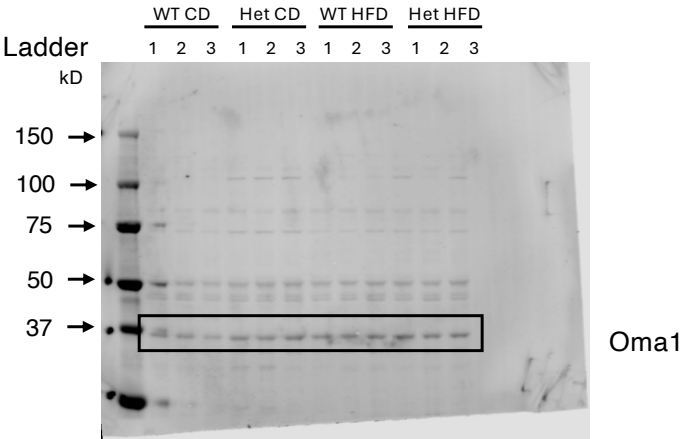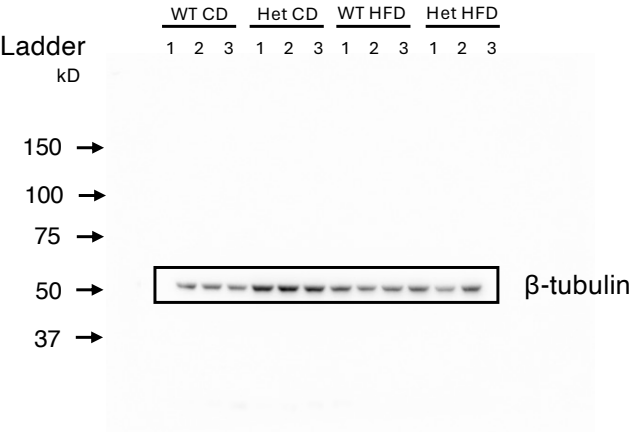

Supplement: Supplementary file 6 — Source data Fig. 8 [file 44321_2024_67_MOESM6_ESM.zip › Figure 8/8N-Q/8P.pdf]

Figure 8J

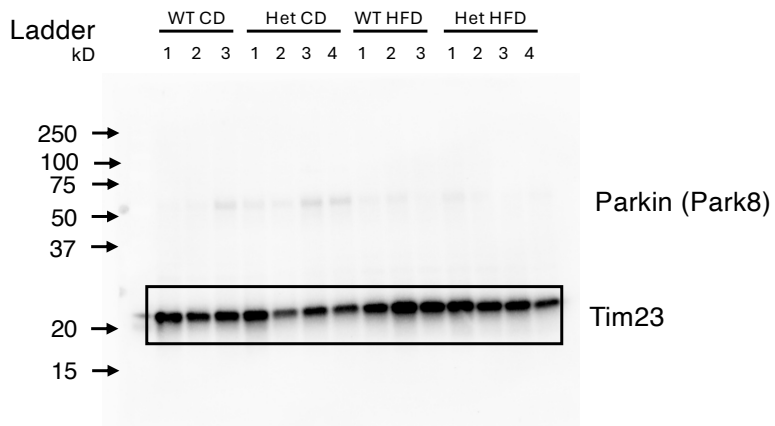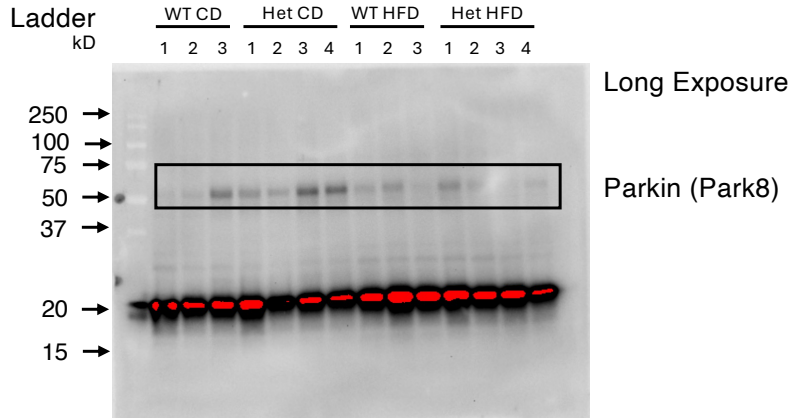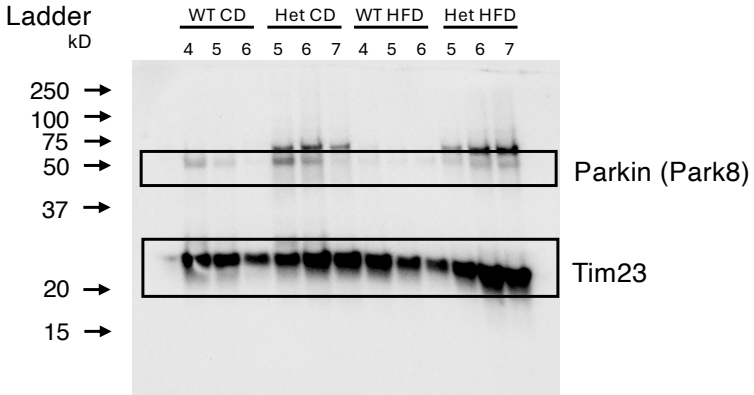

Supplement: Supplementary file 6 — Source data Fig. 8 [file 44321_2024_67_MOESM6_ESM.zip › Figure 8/8J-K/8J.pdf]

Figure 8H

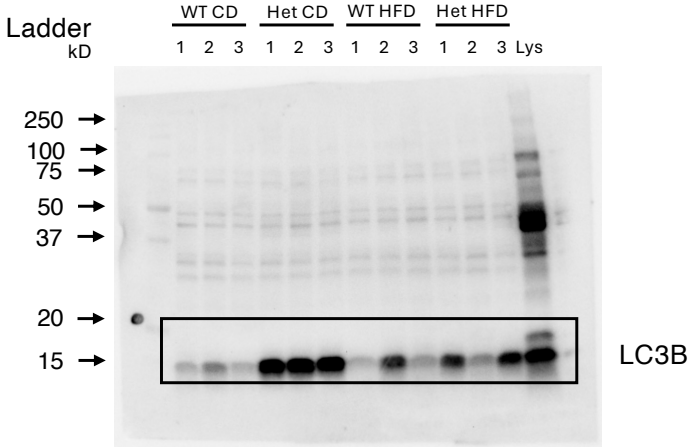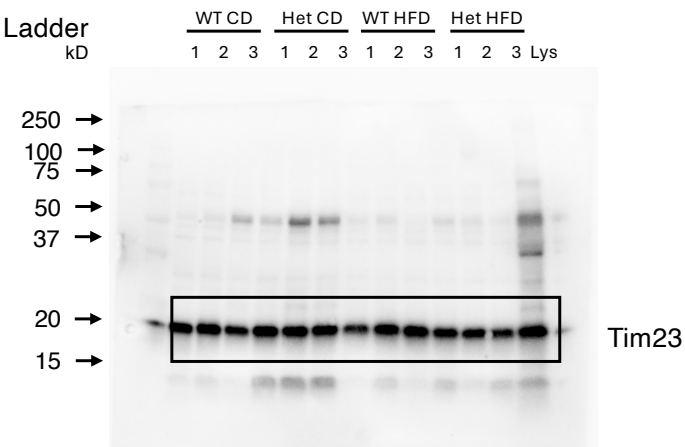

Supplement: Supplementary file 6 — Source data Fig. 8 [file 44321_2024_67_MOESM6_ESM.zip › Figure 8/8H-I/8H.pdf]

Figure 8R

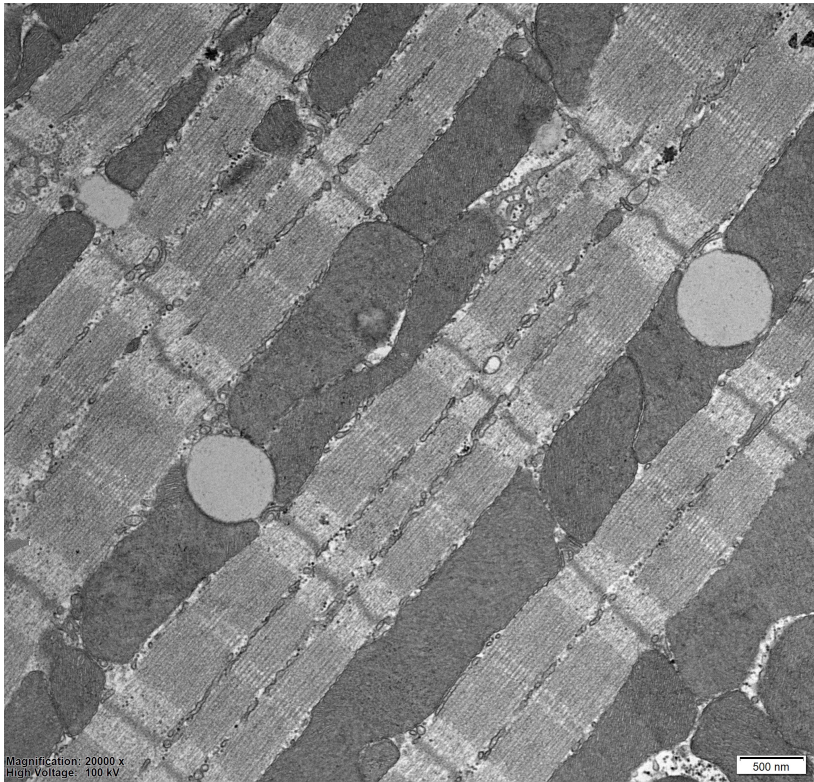

Supplement: Supplementary file 6 — Source data Fig. 8 [file 44321_2024_67_MOESM6_ESM.zip › Figure 8/8R/8R WT HFD.pdf]

Figure 8R

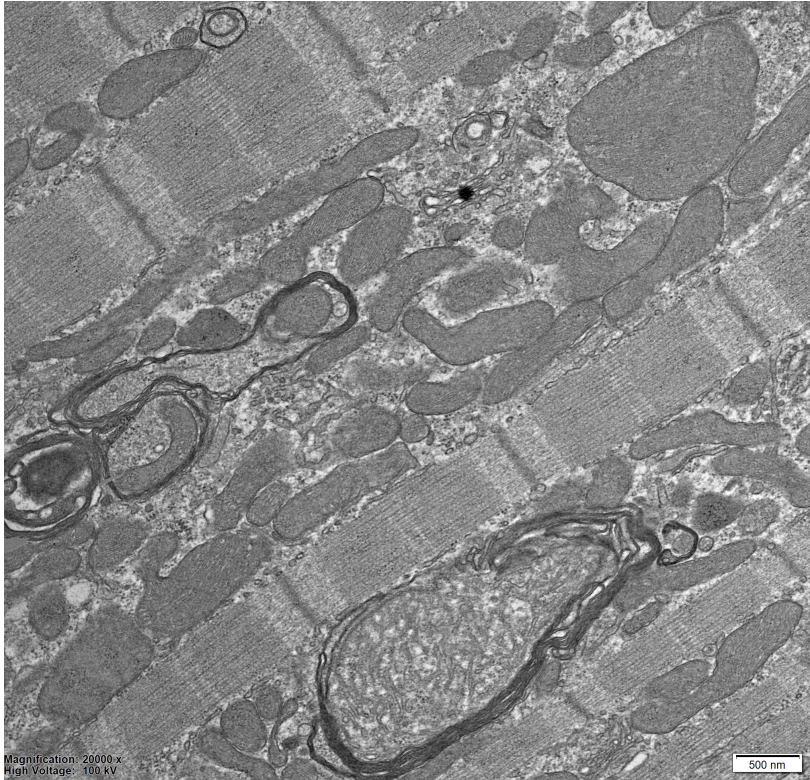

Supplement: Supplementary file 6 — Source data Fig. 8 [file 44321_2024_67_MOESM6_ESM.zip › Figure 8/8R/8R Het HFD.pdf]

Figure 8R

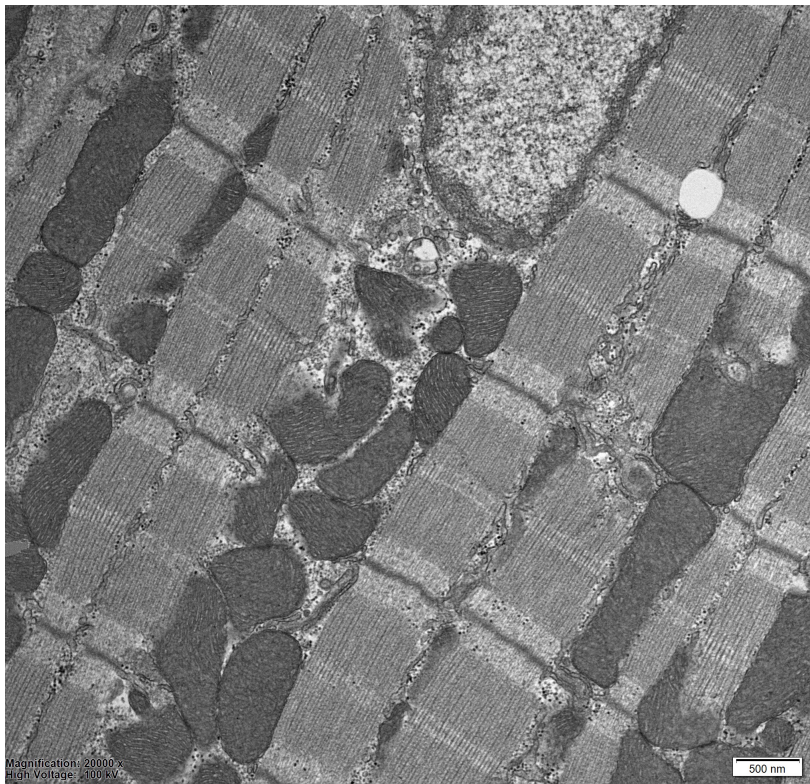

Supplement: Supplementary file 6 — Source data Fig. 8 [file 44321_2024_67_MOESM6_ESM.zip › Figure 8/8R/8R WT CD.pdf]

Figure 8R

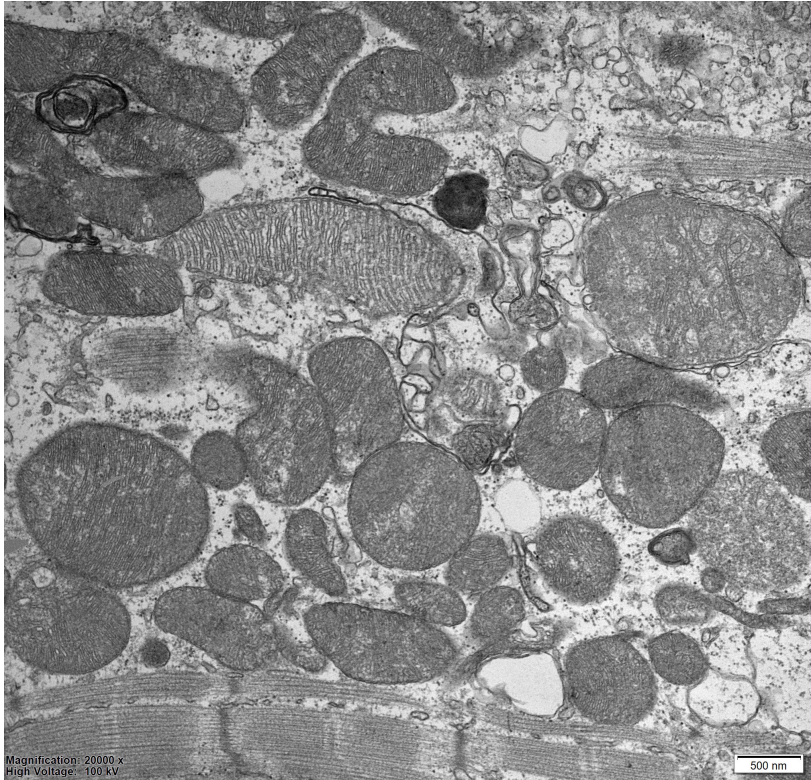

Supplement: Supplementary file 6 — Source data Fig. 8 [file 44321_2024_67_MOESM6_ESM.zip › Figure 8/8R/8R Het CD.pdf]

Figure 8C

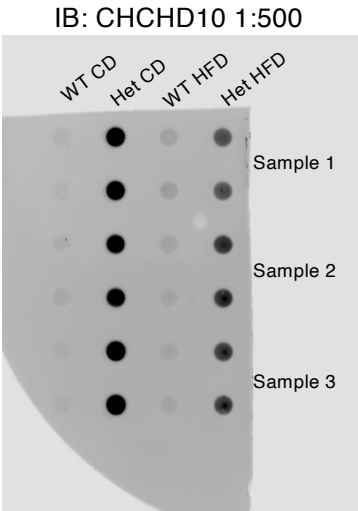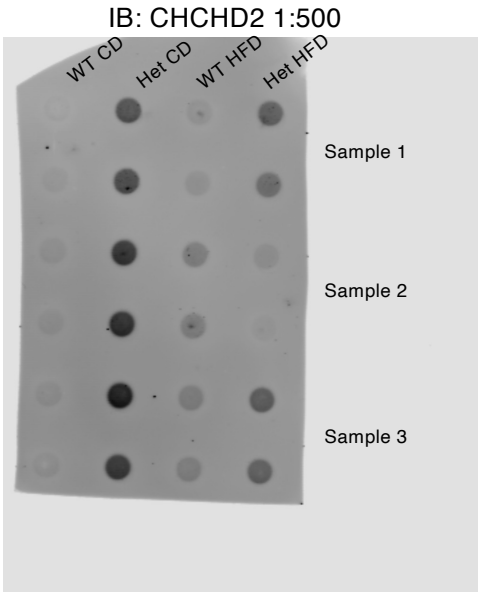

Supplement: Supplementary file 6 — Source data Fig. 8 [file 44321_2024_67_MOESM6_ESM.zip › Figure 8/8C-D/8C.pdf]

Figure 8L

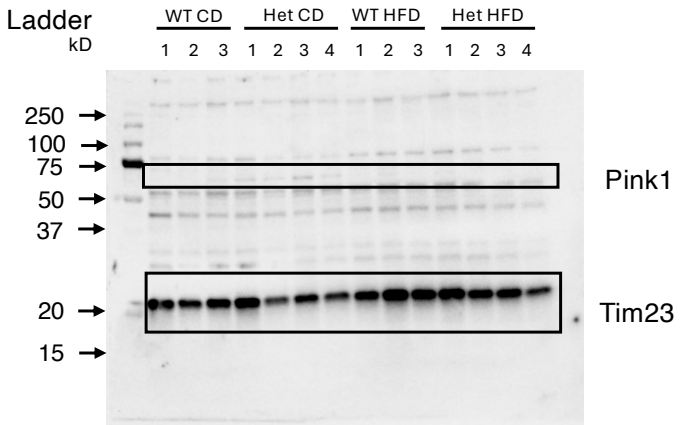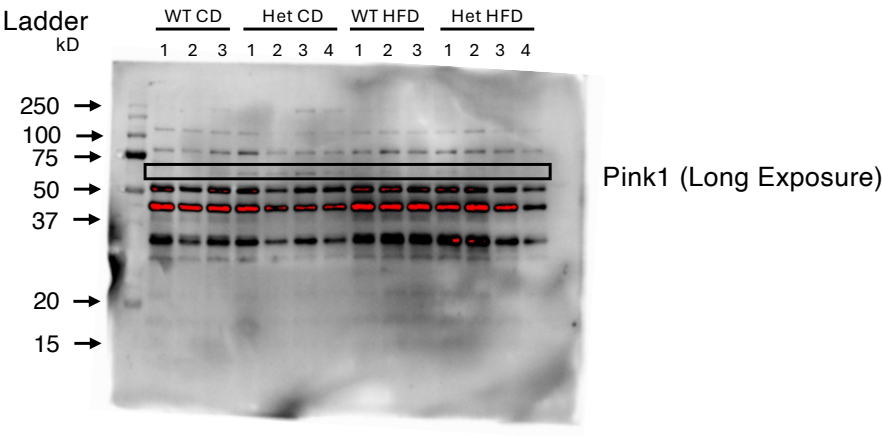

Supplement: Supplementary file 6 — Source data Fig. 8 [file 44321_2024_67_MOESM6_ESM.zip › Figure 8/8L-M/8L.pdf]

Ladder  
kD

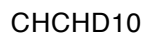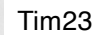

Supplement: Supplementary file 6 — Source data Fig. 8 [file 44321_2024_67_MOESM6_ESM.zip › Figure 8/8A-B/8A.pdf]
