# Supplementary material for: High fat diet ameliorates mitochondrial cardiomyopathy in CHCHD10 mutant mice
Source: EMBO Mol Med. 2024 May 9;16(6):8. doi: 10.1038/s44321-024-00067-5 (PMC11178915; doi:10.1038/s44321-024-00067-5)
Supplement: Supplementary file 9 — Expanded View Figures [file 44321_2024_67_MOESM9_ESM.pdf]

Expanded View Figures

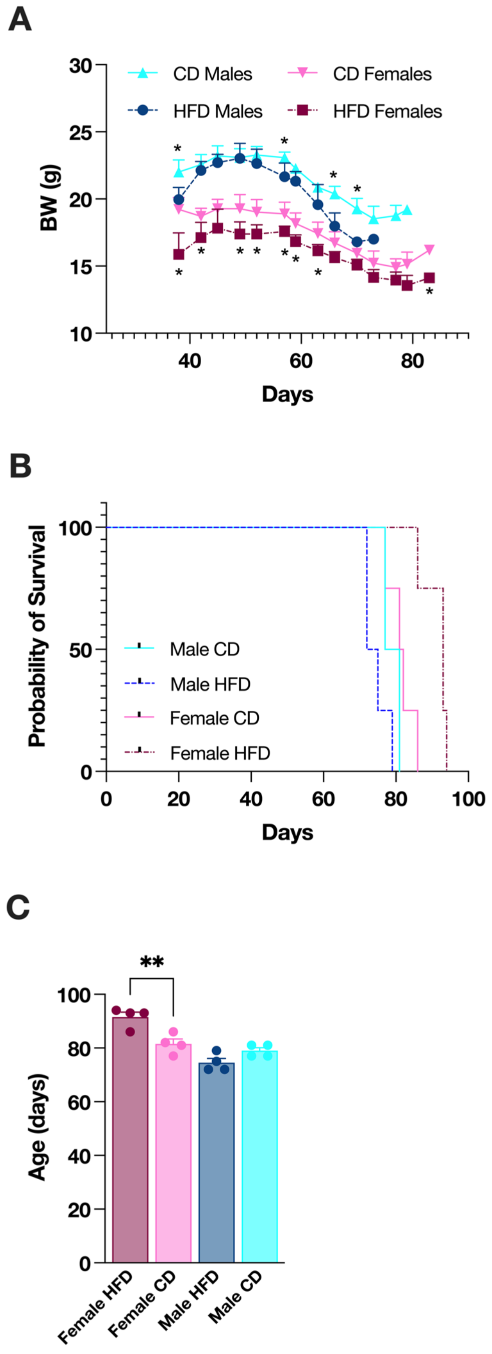

Figure EV1. Effect of HFD in *Fxn* KO mice hearts.

(**A**) Body weight of cardiac *Fxn* KO mice on CD or HFD. (**B**) Kaplan–Meir survival curve of *Fxn* KO mice on CD or HFD. (**C**) Mean age at death of heart *Fxn* KO mice on CD or HFD. Data information: In (**A–C**), *Fxn* KO CD males ($n = 4$ mice), *Fxn* KO CD females ($n = 4$ mice), *Fxn* KO HFD males ($n = 4$ mice), *Fxn* KO HFD females ($n = 4$ mice). For (**A**) and (**C**), statistical significance was determined by unpaired t-tests within each sex. *$p < 0.05$, **$p < 0.01$. Data are expressed as mean ± SEM. Source data are available online for this figure.

© The Author(s)

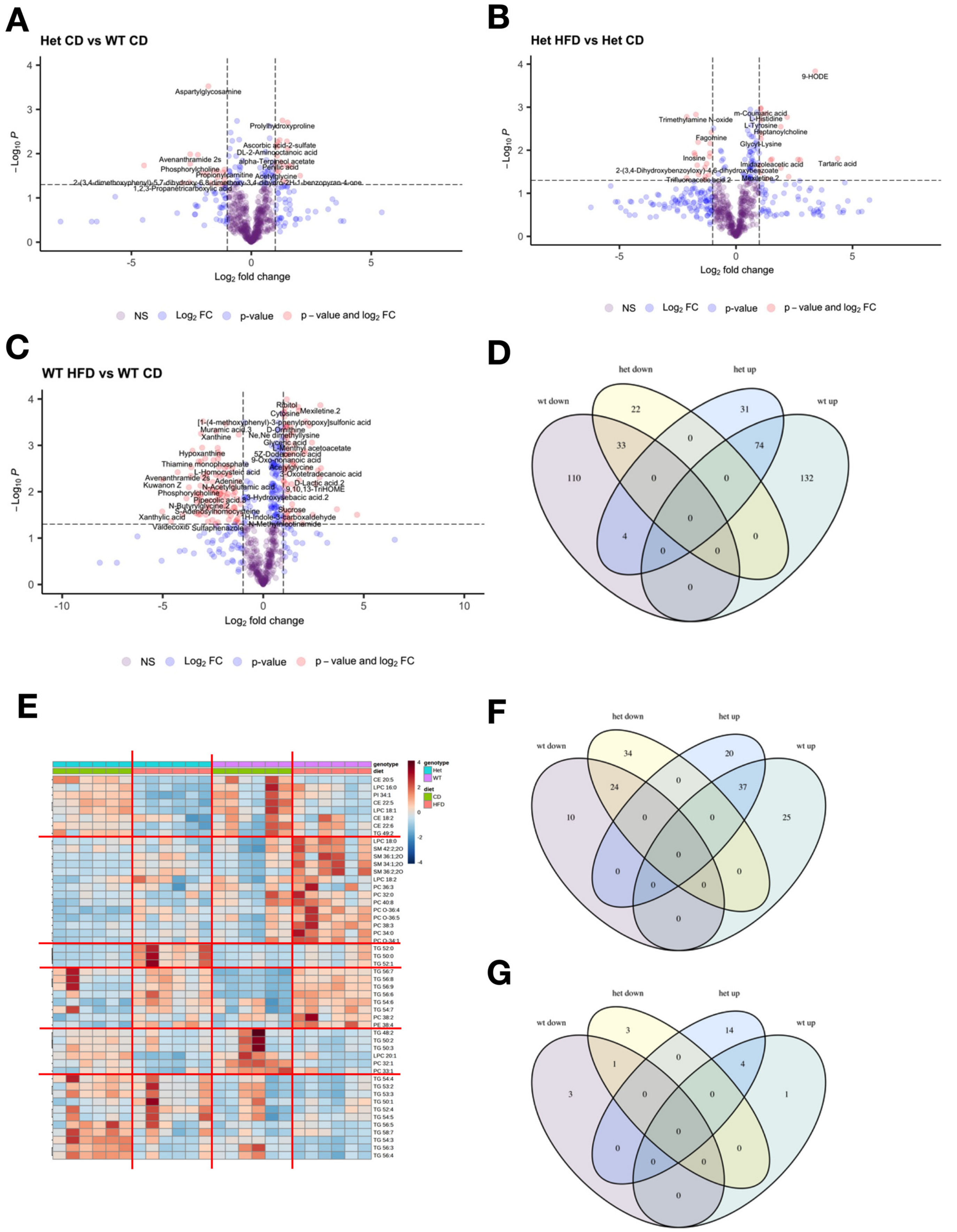
A
Het CD vs WT CD
Aspartylglycosamine
Prolylhydroxyproline
−Log10 P
Log2 fold change
NS
Log2 FC
p-value
p – value and log2 FC
B
Het HFD vs Het CD
9-HODE
Trimethylamine N-oxide
Fagomine
Inosine
Tartaric acid
−Log10 P
Log2 fold change
NS
Log2 FC
p-value
p – value and log2 FC
C
WT HFD vs WT CD
−Log10 P
Log2 fold change
NS
Log2 FC
p-value
p – value and log2 FC
D
wt down
het down
het up
wt up
22
31
33
0
74
110
0
0
132
0
4
0
0
0
0
E
genotype
diet
Het
WT
CD
HFD
F
wt down
het down
het up
wt up
34
20
24
0
37
10
0
0
25
0
0
0
0
0
0
G
wt down
het down
het up
wt up
3
14
1
0
4
3
0
0
1
0
0
0
0
0
0

Figure EV2. Blood metabolomics analyses.

(**A**) Volcano plot of serum metabolites in Het CD and WT CD mice at P250. (**B**) Volcano plot of serum metabolites in Het HFD and Het CD mice. (**C**) Volcano plot of serum metabolites in WT HFD and WT CD mice. (**D**) Venn diagram of serum metabolites both up- and down-regulated by HFD within each genotype separately (Het HFD vs Het CD, WT HFD vs WT CD). (**E**) Heatmap of top 50 metabolites (by interquartile range) in serum of Het and WT mice on CD or HFD. (**F**) Venn diagram of serum lipids up- or down-regulated by HFD within each genotype separately. (**G**) Venn diagram of serum free fatty acids up- or down-regulated by HFD within each genotype separately. Data information: In (**A**–**G**), female groups, WT CD ($n = 6$ mice), Het CD ($n = 6$ mice), WT HFD ($n = 6$ mice), Het HFD ($n = 6$ mice). For (**A**–**C**), the threshold for volcano plots were set at $\log_2 FC = 1$ and $p_{raw} < 0.05$. For (**D**), (**F**) and (**G**), the Venn diagram threshold was $p < 0.1$. For (**E**), the red lines and the numbers on the left denote different groups of metabolites that change based on genotype and diet.

© The Author(s)

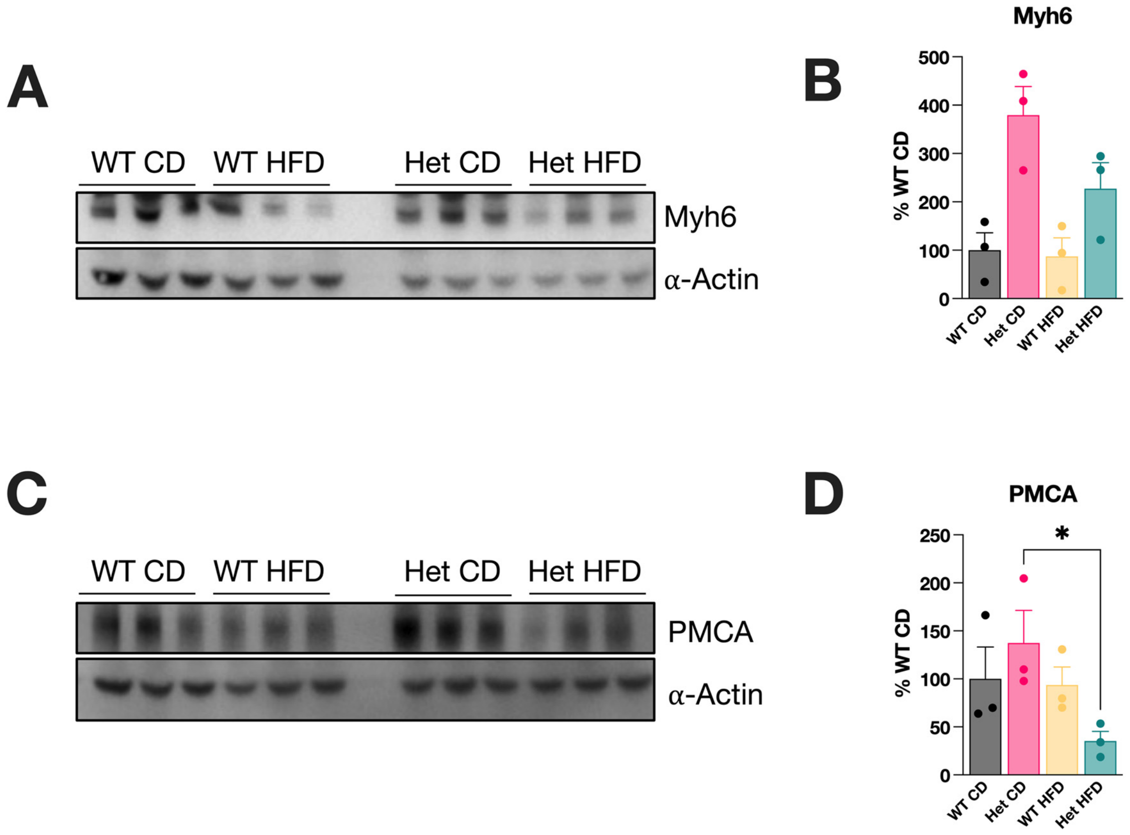

Figure EV3. Decreased protein levels of cardiomyopathy stress markers in Het HFD hearts.

(**A**) Representative western blot image of Myh6 normalized to α-actin. (**B**) Quantification of Myh6 protein levels normalized to α-actin (**C**) Representative western blot image of PMCA (*Atp2b4*) normalized to α-actin. (**D**) Quantification of PMCA protein levels normalized to α-actin. Data information: In (**A–D**), WT CD ($n = 3$ mice), Het CD ($n = 3$ mice), WT HFD ($n = 3$ mice), Het HFD ($n = 3$ mice). For (**B**) and (**D**), data are expressed as mean ± SEM. Statistical significance was determined by unpaired t-test between genotypes. *$p < 0.05$. Source data are available online for this figure.

 © The Author(s)

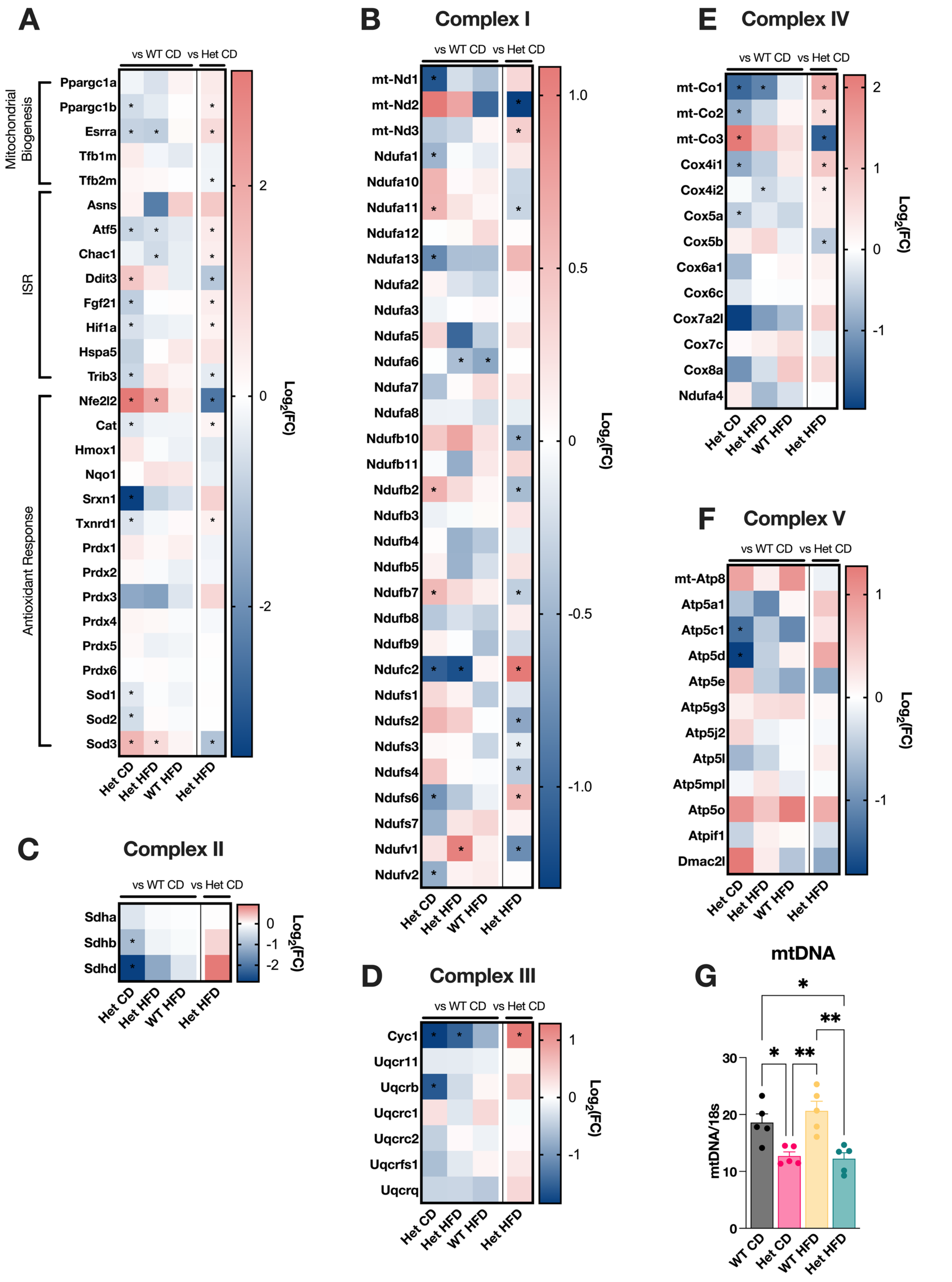
A
vs WT CD vs Het CD
Mitochondrial Biogenesis
Ppargc1a
Ppargc1b
Esrra
Tfb1m
Tfb2m
ISR
Asns
Atf5
Chac1
Ddit3
Fgf21
Hif1a
Hspa5
Trib3
Antioxidant Response
Nfe2l2
Cat
Hmox1
Nqo1
Srxn1
Txnrd1
Prdx1
Prdx2
Prdx3
Prdx4
Prdx5
Prdx6
Sod1
Sod2
Sod3
Het CD Het HFD WT HFD Het HFD
Log2(FC)
B Complex I
mt-Nd1 mt-Nd2 mt-Nd3 Ndufa1 Ndufa10 Ndufa11 Ndufa12 Ndufa13 Ndufa2 Ndufa3 Ndufa5 Ndufa6 Ndufa7 Ndufa8 Ndufb10 Ndufb11 Ndufb2 Ndufb3 Ndufb4 Ndufb5 Ndufb7 Ndufb8 Ndufb9 Ndufc2 Ndufs1 Ndufs2 Ndufs3 Ndufs4 Ndufs6 Ndufs7 Ndufv1 Ndufv2
C Complex II
Sdha Sdhb Sdhd
D Complex III
Cyc1 Uqcr11 Uqcrb Uqcrc1 Uqcrc2 Uqcrfs1 Uqcrq
E Complex IV
mt-Co1 mt-Co2 mt-Co3 Cox4i1 Cox4i2 Cox5a Cox5b Cox6a1 Cox6c Cox7a2l Cox7c Cox8a Ndufa4
F Complex V
mt-Atp8 Atp5a1 Atp5c1 Atp5d Atp5e Atp5g3 Atp5j2 Atp5l Atp5mpl Atp5o Atpif1 Dmac2l
G mtDNA
mtDNA/18s
WT CD Het CD WT HFD Het HFD

Figure EV4. Heart transcriptomics of mitochondrial respiratory complexes, mitochondrial biogenesis, ISR, and antioxidant genes.

(**A**) Heatmap with genes related to mitochondrial biogenesis, ISR, and antioxidant responses. (**B**) Heatmap showing effect of HFD on genes related to Complex I. (**C**) Heatmap with Complex II genes. (**D**) Heatmap with Complex III genes. (**E**) Heatmap with Complex IV genes. (**F**) Heatmap with Complex V genes. (**G**) Heart mtDNA/nDNA (ND5/18s rRNA) ratio by dPCR. Data information: In (**A–F**), WT CD ($n = 10$ mice), Het CD ($n = 10$ mice), WT HFD ($n = 10$ mice), Het HFD ($n = 10$ mice). Equal numbers of female and male mice were studied. Statistical significance was determined by Wald's test. *$p_{adj} < 0.05$. In (**G**), WT CD ($n = 5$ mice), Het CD ($n = 5$ mice), WT HFD ($n = 5$ mice), Het HFD ($n = 5$ mice). Statistical significance was determined by one-way ANOVA with Tukey's correction. *$p < 0.05$, **$p < 0.01$. Data are expressed as mean ± SEM. Source data are available online for this figure.

 © The Author(s)

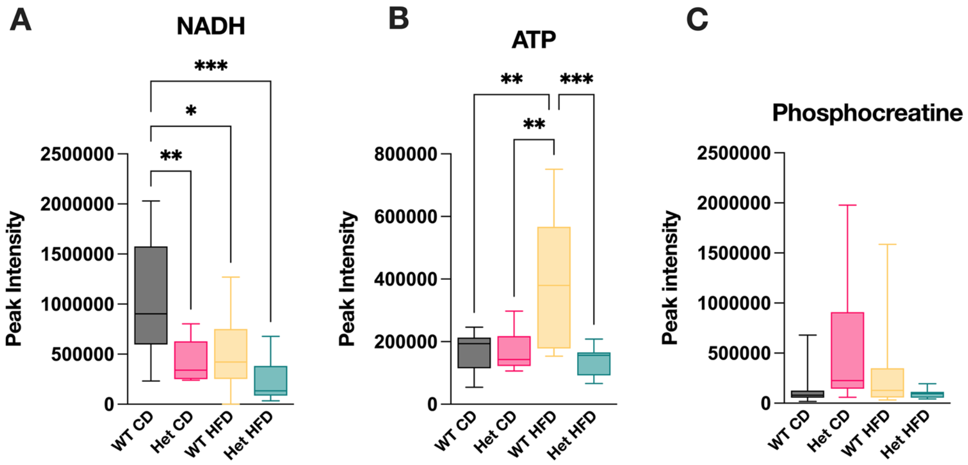

Figure EV5. Intermediate metabolites related to energy status in the heart.

Levels of NADH (**A**), ATP (**B**), and Phosphocreatine (**C**) in heart of Het and WT mice on CD or HFD. Data information: In (**A**–**C**), WT CD ($n = 10$ mice), Het CD ($n = 10$ mice), WT HFD ($n = 10$ mice), Het HFD ($n = 10$ mice). Equal numbers of female and male mice were studied. Statistical significance was determined by one-way ANOVA with Tukey's correction for multiple comparisons. *$p < 0.05$, **$p < 0.01$, ***$p < 0.0005$. Data are expressed as box plots showing minimum to maximum values, median, and upper and lower quartiles. Source data are available online for this figure.
