# Supplementary material for: High fat diet ameliorates mitochondrial cardiomyopathy in CHCHD10 mutant mice
Source: EMBO Mol Med. 2024 May 9;16(6):8. doi: 10.1038/s44321-024-00067-5 (PMC11178915; doi:10.1038/s44321-024-00067-5)
Supplement: Supplementary file 7 — EV and Appendix Figure Source Data [file 44321_2024_67_MOESM7_ESM.zip › EV Figures.zip!/Figure_EV3.zip!/Figure EV3/EV3A-B/Fig EV3A.pdf]

Figure EV 3B

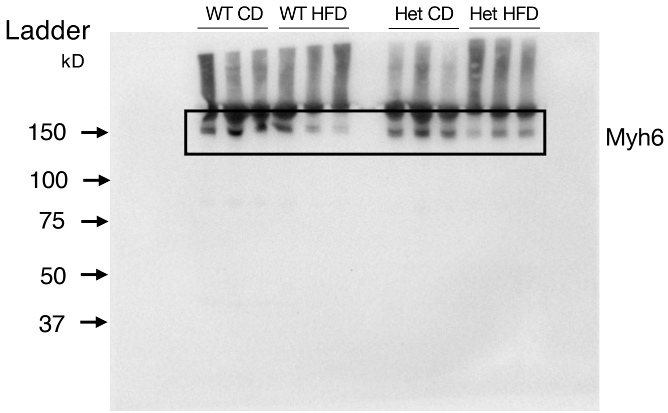

WT CD
WT HFD
Het CD
Het HFD
Ladder
kD
150
100
75
50
37
Myh6

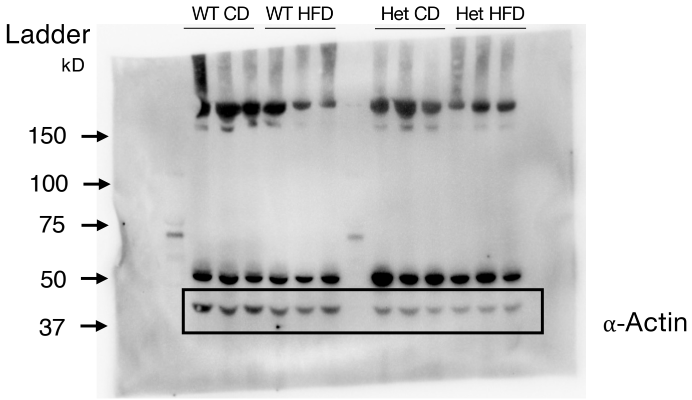

WT CD
WT HFD
Het CD
Het HFD
Ladder
kD
150
100
75
50
37
α-Actin
