# Supplementary material for: High fat diet ameliorates mitochondrial cardiomyopathy in CHCHD10 mutant mice
Source: EMBO Mol Med. 2024 May 9;16(6):8. doi: 10.1038/s44321-024-00067-5 (PMC11178915; doi:10.1038/s44321-024-00067-5)
Supplement: Supplementary file 7 — EV and Appendix Figure Source Data [file 44321_2024_67_MOESM7_ESM.zip › EV Figures.zip!/Figure_EV3.zip!/Figure EV3/EV3C-D/Fig EV3C.pdf]

Figure EV 3C

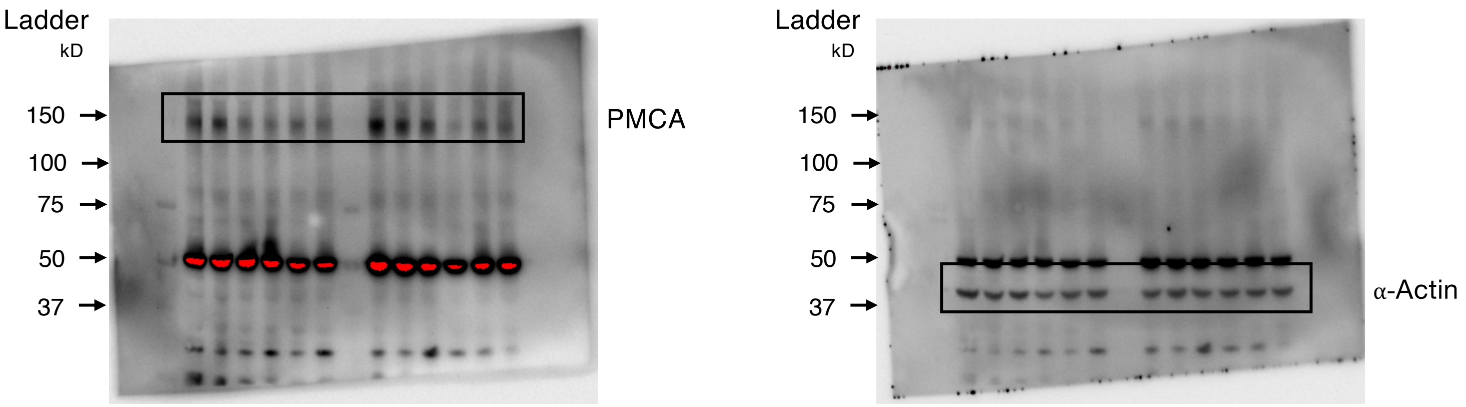

Ladder
kD
150
100
75
50
37
PMCA
Ladder
kD
150
100
75
50
37
α-Actin
